# Redefining bacterial origins of replication as centralized information processors

**DOI:** 10.3389/fmicb.2015.00610

**Published:** 2015-06-16

**Authors:** Gregory T. Marczynski, Thomas Rolain, James A. Taylor

**Affiliations:** Department of Microbiology and Immunology, McGill University, Montreal, QC, Canada

**Keywords:** *oriC*, DnaA, chromosome replication, partitioning, cell-cycle, regulators

## Abstract

In this review we stress the differences between eukaryotes and bacteria with respect to their different cell cycles, replication mechanisms and genome organizations. One of the most basic and underappreciated differences is that a bacterial chromosome uses only one *ori* while eukaryotic chromosome uses multiple *oris*. Consequently, eukaryotic *ori*s work redundantly in a cell cycle divided into separate phases: First inactive replication proteins assemble on eukaryotic *ori*s, and then they await conditions (in the separate “S-phase”) that activate only the *ori*-bound and pre-assembled replication proteins. S-phase activation (without re-assembly) ensures that a eukaryotic *ori* “fires” (starts replication) only once and that each chromosome consistently duplicates only once per cell cycle. This precise chromosome duplication does not require precise multiple *ori* firing in S-phase. A eukaryotic *ori* can fire early, late or not at all. The single bacterial *ori* has no such margin for error and a comparable imprecision is lethal. Single *ori* usage is not more primitive; it is a totally different strategy that distinguishes bacteria. We further argue that strong evolutionary pressures created more sophisticated single *ori* systems because bacteria experience extreme and rapidly changing conditions. A bacterial *ori* must rapidly receive and process much information in “real-time” and not just in “cell cycle time.” This redefinition of bacterial *oris* as centralized information processors makes at least two important predictions: First that bacterial *oris* use many and yet to be discovered control mechanisms and second that evolutionarily distinct bacteria will use many very distinct control mechanisms. We review recent literature that supports both predictions. We will highlight three key examples and describe how negative-feedback, phospho-relay, and chromosome-partitioning systems act to regulate chromosome replication. We also suggest future studies and discuss using replication proteins as novel antibiotic targets.

## Introduction

This short review emphasizes the bacterial point of view for replication control and argues that bacterial chromosome origins (*oris*) of replication have an underappreciated importance for cell cycle control not shared by eukaryotic *oris*. If this view seems controversial, it is not because the data and literature are contradictory. Instead, our view only seems controversial because reviews typically over-emphasize the similarities among organisms. Our presentation aims to restore a balance that respects the complexities of bacteria and eukaryotes. We develop our argument from a historical perspective and then, because space is limited, we give a few specific examples of uniquely bacterial control. Our literature review is therefore incomplete. However, the bacterial cell cycle field is growing and excellent reviews are available to fill the gaps. For example, a very recent review has covered *oris* in diverse model bacteria and it systematically surveyed the many different regulators of replication (Wolanski et al., 2015). The *Escherichia coli oriC* model and the DnaA mechanism for initiating chromosome replication have provided the most detailed molecular mechanisms that operate inside *oris* and recent reviews also provide new insights (Kaguni, 2011; Leonard and Grimwade, 2011; Skarstad and Katayama, 2013; Kaur et al., 2014). An especially lucid review with fine graphic summaries of bacterial cell cycle mechanisms was provided by Katayama and coworkers (Katayama et al., 2010). Our review aims to complement such reviews with a fresh perspective.

## Historical and Theoretical Background

Bacteria were first studied as medical problems and later as simple models or substitutes for complex organisms. Today, bacteria are also studied as interesting organisms in their own right. The three kingdoms view of biology gives bacteria a separate and potentially unique place. Regarding replication genes, we now know that the other two kingdoms, the archaea and eukarya share homologous replication components and it is the bacteria that stand out (Makarova and Koonin, 2013). However, when the replicon hypothesis was first formulated to explain chromosome replication, *E. coli* replication was viewed as a valid and accurate representation for all organisms. This bold assertion reflected the basically valid conviction that all life is united by evolution. However, a unity at the biochemical level does not necessarily imply a unity at higher organizational levels. So while biosynthetic and polymerization reactions may all have common mechanisms, it does not follow that assembly and regulatory reactions should be similarly conserved. How proteins and other cell components bind and sequentially assemble, how these form dynamic cellular structures and how these communicate to regulate cellular functions, are all major themes of contemporary cell biology. We now know that regulatory systems are evolutionarily very flexible and this insight is also expressed in recent bacterial cell cycle reviews (Katayama et al., 2010; Collier, 2012; Jonas, 2014, Wolanski et al., 2015).

Chromosome replication is an especially sophisticated assembly reaction that communicates with many cellular processes. We will argue that bacteria present special challenges and that our studies are far from complete. However, before presenting some contemporary studies, we need to quickly review the original replicon hypothesis, because it has guided and unfortunately also misguided so much of what we know or think that we know.

The replicon hypothesis is now 50 years old (Wolanski et al., 2014). When this hypothesis was first proposed to explain chromosome replication, the operon hypothesis was simultaneously proposed to explain genetic transcription. Both hypotheses were viewed as parallel and complementing explanations for these fundamental processes. For example, while both hypotheses proposed specific DNA targets for proteins, the replicon hypothesis proposed proteins that only acted positively to stimulate DNA synthesis, while the operon hypothesis proposed exclusive negative regulation using the *lac* repressor as the model. In retrospect, it is hard to see why both positive and negative regulators should not have been considered, but this realization would require further studies of the *lac* and other operons as well as studies of RNA polymerase interacting with its promoter DNA sequences. By analogy to transcription promoters, bacterial origins of replication (*oris*) became viewed as places for assembling replication proteins (Kornberg and Baker, 1992). In rough outline, a bacterial *ori* is now viewed as a specific place where the DnaA protein binds multiple DnaA boxes to self-assemble and then to promote the assembly of the downstream replication proteins (Kaguni, 2011; Leonard and Grimwade, 2011; Bell and Kaguni, 2013; Kaur et al., 2014).

## What is the Correct Definition of an Origin of Replication?

Most importantly for this review, the replicon hypothesis gave us the basic concept of “origins (*oris*) of replication.” In other words, an *ori* is a fixed and dedicated place on the chromosome where replication always starts and by analogy to promoters, where most regulators act. While we all take this basic concept for granted, there is in fact no theoretical need for origins of replications as there is for transcriptional promoters. Genetic transcription requires fixed and dedicated promoters to selectively transcribe specific genes so that some genes are “on” while others are “off.” However, if all genes required uniform transcription then specific start and stop sites would be optional and even wasteful. Therefore, to duplicate a whole chromosome the cell does not require that replication always initiates from one fixed place. Instead, what is required is that the chromosome is picked only once for each replication cycle. In fact, this is exactly what eukaryotic cells do in S-phase (Prasanth et al., 2004; Masai et al., 2010). So why do we conventionally say that eukaryotic chromosomes use specific *oris* if they are apparently not needed? This view is primarily a presumption from the earlier bacterial literature. Today, it is more accurate to say that eukaryotic chromosomes use preferential *oris*, including optional and conditional *oris* (Chang et al., 2011) but that they lack the fixed and dedicated *oris* of bacterial chromosomes (Gao et al., 2013). As we will explain further below, eukaryotic chromosomes have preferential *oris* only because the proteins that recognize them (the ORCs, origin recognition complex proteins) have preferential binding sites (Chang et al., 2011). However, the main role of eukaryotic ORC proteins is not to pick the place but the time (S-phase) for replication (Prasanth et al., 2004). ORCs mark the chromosome for replication and ORC placement is much less important. In contrast, the bacterial DnaA protein picks both the time and place to start chromosome replication. This distinction and the special regulatory functions of bacterial *oris* will be more apparent when we next consider the eukaryotic and the bacterial cell cycles.

## Contrasts between Eukaryotic and Bacterial Replication Controls

**FIGURE 1:**
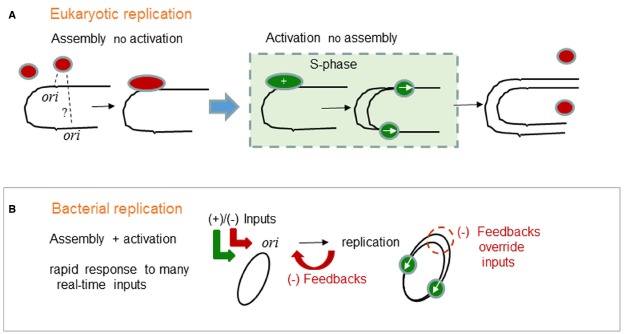
**Generalized logic of (A) eukaryotic and (B) bacterial chromosome replication control. (A)** In eukaryotes, the commitment to chromosome replication occurs at the cellular-level. The whole cell moves into S-phase. Individual eukaryotic *ori*s do not participate in the commitment to S-phase. Instead, eukaryotic *ori*s passively respond to S-phase. Assembly of replication proteins on *oris* is temporally separated from the activation of replication which can only occur once in S-phase. Red ovals are licensed ORC complexes, green ovals are initiation complexes and replication forks started only from those same pre-bound complexes. **(B)** In bacteria, the commitment to chromosome replication occurs at the single *ori-* level. Replication protein assembly and activation are integrated and subjected to many positive and negative (+)/(–) inputs. Precise chromosome duplication, without over-replication, also needs negative (–) feedback mechanisms that transiently override the (+) inputs and block assembly. The green ovals represent active replisomes. Integrated assembly and activation permit rapid and real-time responses that characterize bacterial physiology and permit survival in extreme and in rapidly changing environments.

Eukaryotes and bacteria have very different replication control strategies. In many respects, eukaryotic cell cycle controls are very sophisticated but at the DNA-binding level it is the bacteria that show the sophistication. In eukaryotes, the commitment to chromosome replication occurs at the cellular-level. The whole cell moves into S-phase (Figure 1A). Individual eukaryotic *ori*s do not participate in this commitment, instead they wait and passively respond to global changes such as threshold levels of cyclin-dependent kinases. First, replication proteins assemble on *ori*s and become primed for replication. Another important distinction is the “licensing” concept (Lygerou and Nurse, 2000; Nishitani et al., 2000), because it applies to eukaryotic and not to bacterial chromosomes. Licensing is a protein assembly reaction that occurs in G1 phase. In the separate S-phase only the *or*i-bound “licensed” assemblies can start replication. Assembly of replication proteins on *ori*s and their activation occur in separate phases of the cell cycle. It is this temporal separation that ensures that a chromosome will replicate only once per cell cycle. Precise duplication does not require a precise *ori* response. ORC and licensing proteins need not assemble at every *ori* and every *ori* need not fire (Woodward et al., 2006).

In contrast, bacteria absolutely need a precise *ori* response, because the chromosome has just one *ori*. This fact is unusually misinterpreted as a primitive state compared to eukaryotes. However, bacterial chromosomes are in fact well organized, e.g., the functional unity of operons, and highly evolved compared to those of eukaryotes. The single *ori* is not an accident but an evolved advantage. What advantages does a single *ori* provide? We argue that a single *ori* centralizes information processing. As we summarize for the *ori* in (Figure 1B), bacterial cell cycles do not have well defined phases. Instead, replication protein assembly and activation are integrated and subjected to many positive and negative (++)/(–) inputs (Wolanski et al., 2015). Precise chromosome duplication, without over-replication, also needs negative (–) feedback mechanisms that transiently override the (+) inputs and block assembly (Katayama et al., 2010). Integrated assembly and activation also permits rapid real-time responses that characterize bacterial physiology and permit survival in extreme and in rapidly changing environments.

## Bacterial DnaA Replication Control

The DnaA protein is used by most and possibly all bacteria to initiate chromosome replication (Wolanski et al., 2014) and therefore DnaA is a major target for the positive and negative (+)/(–) *ori* inputs implied schematically in Figure 1B (Wolanski et al., 2015). In *E. coli*, replication begins from a single *oriC* when a critical level of activated DnaA (ATP bound ATP-DnaA) is reached (Katayama et al., 2010; Kaguni, 2011). Both the activated ATP-DnaA and the inactive ADP-DnaA proteins bind to the main DnaA boxes in *oriC*, but only the activated ATP-DnaA proteins will bind and oligomerize at *oriC* using interactions between neighboring AAA^+^ domains (Erzberger and Berger, 2006). Such DnaA assembly causes DNA unwinding and the recruitment of downstream replicative proteins. Specifically, *oriC* DNA unwinding allows DnaA to recruit DnaB, the replicative DNA helicase and DnaC, the helicase loader, onto the single-stranded DNA (Mott and Berger, 2007). Movement of two DnaB hexamers away from *oriC* results in the further recruitment of primase DnaG and the dissociation of the helicase loader DnaC. Next, the DNA polymerase III holoenzyme composed of the Pol III and the β-clamp (DnaN) are recruited to form the “replisome” that synthesizes the complementary DNA strands (Kaguni, 2011; Leonard and Grimwade, 2011; Skarstad and Katayama, 2013; Kaur et al., 2014).

This bacterial initiation process is often compared to eukaryotic entry into S-phase, especially since both DnaA and the ORC proteins use AAA^+^ domains and ATP to facilitate assembly reactions (Erzberger and Berger, 2006). However, there are significant differences with major consequences for replication control. *First*, *E. coli* DnaA assembly at *oriC* is dynamic and *in vivo* there is probably both back and forth assembly and dis-assembly of DnaA until the critical amount of DnaA oligomerization is reached (Leonard and Grimwade, 2011). This is very different than the static licensing factor assemblies that attach to ORC-bound DNA (the eukaryotic *oris*) during G1 and await activation in S-phase. *Second*, the *E. coli* DnaB replicative helicase is loaded during the initiation process that is driven forward by DnaA oligomerization (Bell and Kaguni, 2013). This dynamic loading is also very different than the static replicative helicases (MCM proteins) that pre-loaded on ORC-bound DNA (eukaryotic *oris*) during G1 and await activation in S-phase.

Both dynamic features of *E. coli* replication initiation imply that there are many ways to shift the dynamics of DnaA and DnaB assembly and therefore bacterial initiation has the potential for a rapid response to many regulatory inputs (Figure 1B). In other words, unlike eukaryotic *oris*, the bacterial *oris* have the potential to process many regulatory signals before firing and committing to replication. Also, this processing can happen in real-time, because cell growth is not divided into cell cycle phases. Such regulation is very advantageous, because the conditions for growth and replication can change very rapidly for bacteria. In support of this dynamic view of *ori* signal processing, many regulators have been found and this is a rapidly expanding field of research. However, since recent reviews have covered the many proposed and established regulators of replication (Katayama et al., 2010, Wolanski et al., 2015), we will only present below the control mechanisms that have interested our lab the most. These include the next three topics on negative-feedback control, inputs from two-component systems and the co-regulation of replication with chromosome partitioning.

## Bacterial Negative-feedback Control

The more dynamic bacterial initiation process also creates a greater reliance on negative-feedback controls. In eukaryotes, the licensing mechanisms automatically quench extra replication from the same *ori* in S-phase. In bacteria, as implied schematically in Figure 1B, to avoid potentially lethal over-replication, negative feedbacks must quench the forward replication potential created by high levels of active ATP-DnaA. *E. coli* has several negative-feedback mechanisms but the dominant one uses DnaN as a key regulatory component (Camara et al., 2003). DnaN forms a ring around the DNA to hold Pol III at the replication forks and a new DnaN ring is formed at each Okazaki fragment. Once replication starts, surplus DnaN rings accumulate and provide a platform for negative feedback regulators that limit replication. In *E. coli* this major regulatory mechanism of inhibiting replication is called RIDA for regulatory inactivation of DnaA. RIDA promotes ATP hydrolysis of ATP-DnaA and thus increases the ratio between inactive ADP-DnaA and active ATP-DnaA in the cell. Hda binds the DnaN ring which slides on the DNA to bring Hda into contact with DNA-bound DnaA protein. Hda has an AAA^+^ domain that contacts the homologous AAA^+^ oligomerization domain on DnaA and this is the specific interaction that stimulates the hydrolysis of DnaA-bound ATP (Kato and Katayama, 2001; Katayama et al., 2010; Nakamura and Katayama, 2010). Since ADP-DnaA cannot oligomerize, Hda can be regarded as an anti-oligomerization or as an anti-DnaA assembly factor.

If the *E. coli oriC* model applies to most bacteria and if surplus DnaN rings are deposited when replication starts, then do other bacteria also use RIDA? Yes, there is good evidence that the distantly related Gram-negative *Caulobacter crescentus* also uses a RIDA-like system. The *C. crescentus* homolog HdaA is very similar to *E. coli* Hda, and as expected down-regulation of HdaA causes chromosome over-replication (Collier and Shapiro, 2009). Also, fluorescence resonance energy transfer experiments demonstrate that *C. crescentus* HdaA interacts with DnaN in live cells (Fernandez-Fernandez et al., 2013). However, unlike *E. coli* DnaA protein, the *C. crescentus* DnaA protein is also regulated by cell cycle proteolysis (Gorbatyuk and Marczynski, 2005; Jonas et al., 2013). Therefore, it is important to consider that HdaA may regulate DnaA through both of these mechanisms and thereby fine-tuning DnaA activity more precisely for a cell cycle program which under natural conditions will experience sudden changes of nutrients, antibiotics and other growth challenges.

In distantly related Gram-positive *Bacillus subtilis*, a negative feedback system similar to RIDA is also present but it certainly evolved independently (Noirot-Gros et al., 2002, 2006). In this system, Hda is replaced by YabA. Interestingly, despite the lack of homology, YabA still forms a stable complex with DnaA as well as with DnaN. Deletion or mutations in *yabA* cause severe over-initiation of chromosome replication and *yabA* over-expression inhibits replication (Noirot-Gros et al., 2002; Goranov et al., 2009). Localization experiments also shown that YabA is associated with the replisome during chromosome replication through its interactions with DnaN (Goranov et al., 2009). Both YabA and Hda have been interpreted as anti-cooperativity or anti-assembly factors that block the critical DnaA oligomerization step on *oriC* (Merrikh and Grossman, 2011).

## Bacterial *ori* Regulation by Two-component Systems

The two-component systems proteins are an especially important class of regulators. These proteins dominate bacteria adaptive responses probably because they have a modular organization that aids the rapid evolution of paralogs that are easily altered to transduce many different signals (Garcia Vescovi et al., 2010; Capra and Laub, 2012). A conserved histidine kinase (HK) module and a conserved a response regulator (RR) module form the basis of a two-component signaling system. Although there is much variety, in many systems the HK is linked to a receptor while the RR is linked to a DNA-binding domain and the HK phosphorylates its cognate RR thereby sending the signal for activating the RR protein.

The *C. crescentus* RR protein called CtrA was the first example of bacterial *ori* regulation by a two-component system (Quon et al., 1996, 1998). Given the ubiquity and adaptive value of two-component systems, their regulatory inputs should be both common and varied. Since the first reports on CtrA, other RR proteins have been reported to regulate or at least to bind inside bacterial *oris*. Such examples include ArcA in *E. coli* (Lee et al., 2001), MtrA in *Mycobacterium tuberculosis* (Rajagopalan et al., 2010), Spo0A in *B. subtilis* (Boonstra et al., 2013), and most recently HP1021 in *Helicobacter pylori* (Donczew et al., 2015). In each case, the RR probably co-regulates replication with global cell activities, because each regulates many genes and the targets inside the *ori* are few compared to the many targets in the whole genome. Regarding the global cell activities, these probably include co-regulation with anaerobic growth by ArcA, macrophage invasion by MtrA, starvation-induced sporulation by Spo0A and stomach colonization by HP1021. Therefore, in each of these cases, environmental signals that drastically affect cell physiology are shunted into the *ori* for information processing, i.e., interactions with other replication proteins. In most cases these inputs are negative. For example, *E. coli* ArcA binds and blocks *ori* unwinding while *H. pylori* HP1021 probably binds to exclude DnaA from *ori*. However, these mechanisms of action are inferred primarily from *in vitro* studies and the *in vivo* activities are probably more complex.

CtrA remains the best studied example of bacterial *ori* regulation by two-component systems. CtrA (cell cycle transcription regulator) as the name implies regulates many cell cycle processes including DNA methylation and cell division (Quon et al., 1996; Kelly et al., 1998). CtrA is an essential master regulator of the dimorphic cell cycle that characterizes *C. crescentus* and therefore CtrA links chromosome replication with a series of intrinsic cell cycle programs that direct cell development.

**FIGURE 2:**
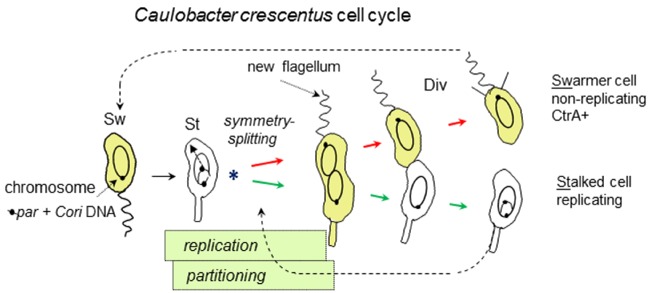
**Asymmetric cell division of *C. crescentus*, emphasizing key events and overlapping chromosome replication and partitioning periods.** Swarmer cells (Sw) differentiate into stalked cells (St) and start chromosome replication with asymmetric (Sw and St-polar) division (Div). The chromosome origin of replication (*Cori*) initiates replication only once in the St cells. Linkage to the partition operon (*par*, containing *parABS*) ensures *Cori* placement at opposite cell poles. The asterisk (*) marks the chromosome symmetry-splitting stage of chromosome partitioning that is described in the text. CtrA protein (yellow) tracks the Sw cell-type due to its cell cycle synthesis and proteolysis.

Understanding CtrA regulation requires the following outline of the *C. crescentus* cell cycle (Figure 2): The non-replicating swarmer cell-type swims until it differentiates into the replicating stalked cell-type. Chromosome replication initiates only once in the stalked cell-type (Marczynski, 1999) which proceeds to grow and divide asymmetrically such that a new swarmer cell-pole is built opposite to the stalked cell-pole. Once replication initiates, the newly replicated DNA is partitioned into these emerging cell compartments that upon cell division will become distinct replicating (stalked) and non-replicating (swarmer) cell-types. CtrA activity is associated with the swarmer cell-type and although CtrA has multiple roles, a major role is to bind and repress the *C. crescentus* origin of replication (*Cori*) in the non-replicating swarmer cells (Quon et al., 1998; Siam et al., 2003; Bastedo and Marczynski, 2009).

How is CtrA activity regulated? This complex topic itself requires a separate review (Tsokos and Laub, 2012). For our purposes, we note that synthesis and proteolysis adjust CtrA protein concentrations so that they are high in swarmer but low in stalked cells. However, protein turn-over is a secondary layer of regulation and as expected, CtrA activity is primarily adjusted by phosphorylation of its cognate RR domain (Domian et al., 1997; Spencer et al., 2009). The dimorphic and asymmetric mode of cell division directs CtrA phosphorylation through kinases and phosphatases that are localized at the swarmer and stalked cell poles (Tsokos and Laub, 2012). It is misleading to call this a “two-component” system, because like Spo0A of *B. subtilis*, CtrA activity is the final readout of a phopho-relay system that integrates many signals with multiple HK and RR modules. Such phosphor-relays do not just pass the signal, they in effect “decide” whether or not to pass the signal by in effect “consulting” many lateral inputs. One interesting aspect of the *C. crescentus* phopho-relay is that it creates a spatial gradient of CtrA activity during asymmetric cell division from high CtrA activity at the emerging swarmer cell-pole to low CtrA activity at the stalked pole (Chen et al., 2011). Another, very interesting aspect of the CtrA phospho-relay is a novel compartment sensing mechanism, so that once the compartments seal, the communication between the opposite poles is cut and this in turn strongly increases CtrA activity in the swarmer compartment while CtrA activity is quenched in the stalked cell compartment (Childers et al., 2014).

How does the *C. crescentus* origin of replication (*Cori*) use CtrA? *Cori* has five high-affinity binding sites for CtrA (Siam and Marczynski, 2000) and four of these sites are evolutionarily conserved among freshwater *Caulobacter* species (Shaheen et al., 2009). Interestingly, the *oris* of some marine *Caulobacter* species also use CtrA but unexpectedly, this usage probably evolved independently. *Caulobacters* belong to the alpha-proteobacteria and while CtrA seems to be a master regulator in this whole group of bacteria (Brilli et al., 2010), except possibly for *Rickettsia prowazekii* (Brassinga et al., 2002), CtrA binding sites are not seen in other *oris*. Therefore, CtrA also illustrates the principle that regulatory systems are evolutionarily very flexible.

What mechanisms does CtrA use to regulate *Cori*? One mechanism may involve transcriptional promoter activation in the stalked cells (Siam and Marczynski, 2000), but how new RNA synthesis promotes replication is not yet clear. The simplest mechanism seems to be a steric exclusion of DnaA protein from *Cori* (Taylor et al., 2011). Therefore, when CtrA activity rises in swarmer cells it binds and blocks replication in the swarmer cells by excluding DnaA. Interestingly, *Cori* has two classes of DnaA binding sites: A moderate affinity class termed G-boxes and a very weak class termed W-boxes (Taylor et al., 2011). The G-boxes have a conserved T to G substitution that reduces the otherwise high affinity of typical DnaA boxes present in other bacterial *oris*. *Cori* has only two G-boxes and both are targeted by their proximity or overlap with CtrA binding sites. The W-boxes are very weak and require cooperative binding with G-boxes for occupancy. The relatively weak G-box and W-box binding sites seem to have a precisely tuned low affinity for DnaA, because mutations that increase their affinity for DnaA can unexpectedly decrease replication (Taylor et al., 2011).

Therefore, *Cori* presents what seems to be a contradiction. *Cori* has a high affinity for CtrA (a protein not typically associated with *oris*) and yet a relatively low affinity for DnaA (the protein that is always required for bacterial *ori* function). In fact *Cori* is the highest affinity target for CtrA in the whole genome (Laub et al., 2002; Taylor et al., 2011). In contrast, since DnaA is also a transcription regulator, many *C. crescentus* promoters have DnaA boxes and some have higher affinity DnaA boxes than those in *Cori* (Hottes et al., 2005; Taylor et al., 2011). To better understand how CtrA binding regulates *Cori*, we systematically removed the CtrA binding sites from *Cori* at its natural locus on the chromosome (Bastedo and Marczynski, 2009). By combining site-directed mutations with homologous recombination, we created strains with substantially lower CtrA affinity in all five binding sites. To our surprise, the normal cell cycle program of chromosome replication was only mildly perturbed. Our interpretation of this result is that under constant laboratory culture conditions, the cell cycle runs like a clock. Most likely DnaA regulators and particularly RIDA (as discussed above) drive the replication cycle with only small adjustments form CtrA (Jonas et al., 2011). Such results forced us to reconsider *Cori* regulation, because obviously *C. crescentus* did not evolve in laboratory cultures but faced many environmental stresses that required constant monitoring. Typical environmental stresses for *C. crescentus* might be starvation and antibiotics. To support this view, we noticed that *C. crescentus* strains lacking CtrA binding at *Cori* became very sensitive to otherwise sub-lethal pulses of antibiotics (Bastedo and Marczynski, 2009).

Most significantly, *Cori* CtrA binding sites become essential when cells encounter both nutrients and antibiotics, a situation that presumably simulates natural bacterial competition and evolutionary pressures (Bastedo and Marczynski, 2009). Therefore, CtrA has at least two major roles in *Cori*: *First*, to help maintain or reinforce the cell cycle pattern of replication, so that replication is “off” in swarmer cells and “on” in the stalked cells (Figure 2). *Second*, to coordinate replication with cell growth in stressful and rapidly changing environments (nutrient up-shifts and antibiotic pulses). We argue that it is this second role for CtrA that provided the main selective pressure for evolving control by CtrA. This second role also presumes rapid real-time inputs into *Cori* that target DnaA. We tentatively interpret the G-box and W-box distribution in *Cori* (Taylor et al., 2011) as a variation of the DnaA box distribution in *E. coli oriC* that permits dynamic back and forth assembly and dis-assembly of DnaA (Leonard and Grimwade, 2011) until regulatory inputs, from CtrA and probably other regulators, drive the DnaA oligomerization toward critical initiation levels. Our search for additional *Cori* regulators identified a novel protein termed OpaA that we describe below, because it participates in both chromosome replication and partitioning. In addition to real-time inputs, environmental signals, such as sudden starvation, are especially important to arrest the normal clockwork cell cycle pattern. For example, such arrests happen when *C. crescentus* is starved and DnaA is removed by targeted proteolysis (Gorbatyuk and Marczynski, 2005; Lesley and Shapiro, 2008; Jonas et al., 2013). Limited space does not allow us to expand on this topic, but the importance of environmental signals for bacterial cell cycle regulation as well as some recent developments have also received a fine review (Jonas, 2014).

## Co-regulation of Chromosome Replication and Chromosome Partitioning

The initiation of chromosome replication immediately precedes the initiation of chromosome partitioning into the daughter cell compartments that will eventually form the daughter cells at cell division (Toro and Shapiro, 2010; Figure 2). This close temporal link suggests that it would be advantageous to co-regulate replication and partitioning. In many bacteria, chromosome partitioning employs a tripartite Par system consisting of a chromosomal centromere site (*parS*), a DNA binding protein (ParB) that binds *parS* DNA and a Walker-type ATPase protein (ParA) that probably uses non-specific DNA sequence affinity and ATP hydrolysis to pull the ParB-*parS* complex into opposite daughter compartments (Vecchiarelli et al., 2010). Interestingly, the *parS* site is usually located close to the *ori*, presumably to minimize the delay between replication and the onset of chromosome partitioning. For example, in *C. crescentus* the *parS* site is located within 8 kb of *Cori*, and in *B. subtilis* the three primary *parS* sites are located within 10 kb of *oriC*. In a survey of over 1,000 genomes, 92% of the *parS* sites were found to be located in the 15% of the chromosome closest to the *ori* (Livny et al., 2007).

Given these close temporal and spatial links, what is the evidence for co-regulation and communication between the replication and partitioning systems? In *B. subtilis*, Soj (a ParA homologue) directly interacts with DnaA protein to regulate replication both positively and negatively at *oriC*, depending on the quaternary state of Soj protein (Murray and Errington, 2008). In turn, Spo0J (ParB homologue) regulates this quaternary state, thus controlling replication through Soj (Scholefield et al., 2011). An innovative study employing recombinant DnaA to allow specific crosslinking of DnaA molecules during their helical oligomerization showed that monomeric-Soj/DnaA interaction blocks the formation of helical DnaA oligomers both *in vivo* and *in vitro* (Scholefield et al., 2012). The mechanism by which dimeric Soj positively influences replication remains unclear but these studies clearly establish co-regulation.

*Vibrio cholera* provides more insights from a very different evolutionary perspective. Unlike most bacteria, *V. cholera* has two chromosomes that use different replication-initiation mechanisms. Chromosome I (chrI) encodes and employs the canonical DnaA mediated replication mechanism while chromosome II (chrII) encodes and employs a different protein, RctB, which performs the analogous initiation function (Egan and Waldor, 2003). Both chromosomes also encode their own Par systems, which act specifically on the chromosome that encodes them. Most interestingly, both Par systems also regulate the replication of their respective chromosomes. ChrI replication is stimulated by ParA1, apparently through direct interactions with DnaA, while ParB1 plays an inhibitory role (Kadoya et al., 2011). On chrII, where replication is initiated by the RctB protein, titration of RctB by the *rctA* site, adjacent to the *ori*, inhibits replication (Venkova-Canova et al., 2006). Yamaichi and colleagues showed that this inhibition is counteracted by ParB2 binding to a *parS2* site within the *rctA* site (Yamaichi et al., 2011). In addition, ParB can directly compete for a strong RctB binding site that inhibits replication within *oriCII* (Venkova-Canova et al., 2013). Thus two ParB2 activities promote replication by reducing RctB binding to inhibitory DNA sequences. These results suggest co-regulation whereby replication is promoted only when ParB2 levels become sufficient for chromosome partitioning.

The previous examples show how partitioning systems can signal replication initiation but logically the signals could flow both ways. Accordingly, a recent study by Mera and colleagues implicated DnaA in controlling ParA dependent chromosome partitioning in *C. crescentus* (Mera et al., 2014). A conditional DnaA expression strain, in which DnaA was shut off failed to initiate chromosome replication, as expected (Gorbatyuk and Marczynski, 2001), and kept the single ParB/*parS* centromere complex at the old cell pole. However, when DnaA was expressed at a low concentration that was insufficient to initiate replication, some cells “partitioned,” i.e., moved the single un-replicated ParB/*parS* centromere complex to the new cell pole using the ParA mechanism. This faulty partitioning requires a DnaA binding site located within *parS*, suggesting that DnaA binding at *parS* directly controls partitioning.

Closer examination of *C. crescentus* chromosome partitioning suggests a need for novel components and perhaps novel mechanisms at the earliest stage of chromosome partitioning. This is a key chromosome symmetry-splitting stage (Figure 2), because immediately following the start of chromosome replication one *parS* locus will stay at the staked pole while the other *parS* locus will partition to the swarmer pole. Subsequent replication will eventually yield polarized chromosomes in their respective stalked cell (replicating) and swarmer cell (non-replicating) compartments (Figure 2). Time-lapse microscopy showed that this partitioning is a multi-step process involving *parS* separation, *parS* discrimination, *parS* slow-movement away from the stalked pole and finally *parS* fast-movement toward the swarmer pole (Shebelut et al., 2010). Further genetic analysis showed that only the final *parS* fast-movement step requires ParA (Shebelut et al., 2010). Therefore, neither the regulators nor the motors of the preceding early steps are known. However, we can speculate that as for DnaA (described above) novel partitioning components might be found among the proteins that first interact with the origins of chromosome replication. These considerations also provide a further motivation for seeking novel replication proteins.

Therefore, co-regulation of partitioning and replication control systems is both phylogenetically widespread and diverse in terms of the molecular interactions involved. Such co-regulation may be advantageous as it ensures that protein concentrations or activity levels required for each process are achieved simultaneously. To our knowledge, no studies have systematically addressed whether the proximity of *par* and *ori* loci is also important for their co-regulation. However, the conservation of this proximity among so many bacterial chromosomes argues very strongly that *par* and *ori* communication is an important part of uniquely bacterial cell cycle strategies.

## Implications for Novel Antibiotic Targets

We are running out of antibiotics and options for treating antibiotic-resistant infections. This fact is well known but if history is any guide, then new treatments will probably not come from established studies but from unexpected sources revealed by new basic research. Chromosome replication studies will contribute toward finding new antibiotics for at least two major reasons: *First*, because replication is essential and it predisposes cells to lethal damage; *Second*, as we argued in this review, because replication must communicate with essential cell cycle processes including for example chromosome partitioning. The first reason suggests finding new direct targets for antibiotics that might disrupt replication regulators. While the second reason suggests that indirect targets may be equally valuable. Such targets may not be directly lethal but they could nonetheless be very effective as *in vivo* antimicrobials.

This short review cannot begin to address this question but it again raises our main issue of bacterial molecular communication and our reinterpretation of *oris* as centralized information processors. From the microbe’s point of view, an infection requires complex navigation and communication in an ever-changing, alternatively hostile and benign tissue environment. As we argue, such communication must ultimately connect with *ori* which must process much information in real-time to determine the life or death of the cell. Therefore, an effective *in vivo* antimicrobial may be one that confuses bacteria so that they make mistakes and fall prey to the natural and overwhelming antimicrobial activities of the immune system. Finding such targeted antimicrobials requires much better knowledge of bacterial communication. Given the varieties of bacterial communication, it is also likely that future antibiotics may be customized for the specific regulators of specific species. We normally think of personalized medicine as a match between a specific human genotype and a specific medication. In the future, considering the ease of identifying bacteria by deep-sequencing techniques, another form of personalized medicine may be a matching between a microbial genotype and specific replication-disrupting antibiotics.

### Conflict of Interest Statement

The authors declare that the research was conducted in the absence of any commercial or financial relationships that could be construed as a potential conflict of interest.
